# AN INFLAMMATORY SIGNATURE OF POSTOPERATIVE DELIRIUM

**DOI:** 10.1093/geroni/igz038.3027

**Published:** 2019-11-08

**Authors:** Sarinnapha Vasunilashorn, Long H Ngo, Simon Dillon, Hasan Otu, Bridget Tripp, Sharon Inouye, Towia A Libermann, Edward R Marcantonio

**Affiliations:** 1 Beth Israel Deaconess Medical Center, Boston, Massachusetts, United States; 2 University of Nebraska-Lincoln, Lincoln, Nebraska, United States; 3 Harvard Medical School Aging Brain Center, Boston, Massachusetts, United States

## Abstract

Delirium is a common, morbid, and costly geriatric syndrome, yet its pathophysiology remains poorly understood. In a nested matched case-control study within the Successful Aging after Elective Surgery (SAGES) study, a cohort of adults age ≥70 without dementia undergoing major non-cardiac surgery, we previously identified inflammatory proteins to be associated with delirium. Using the entire SAGES cohort, the current study examines the independent associations of these inflammatory proteins with postoperative delirium. Plasma was collected preoperatively (PREOP) and on postoperative day 2 (POD2). Neuroinflammatory marker chitinase-3-like protein [CHI3l1 or YKL-40]; PREOP and POD2) and systemic inflammatory markers interleukin [IL]-6 (POD2 only) and C-reactive protein (CRP; PREOP and POD2) were measured using enzyme-linked immunosorbent assays. Generalized linear models were used to determine the independent (multivariable) associations between the inflammatory markers, measured in sample-based quartiles (Q). All models adjusted for age, sex, baseline cognition, surgery type, Charlson comorbidity index, and medical complications. Among the 555 patients (mean age 77 years, standard deviation, SD 5.2), 58% were female and 86% underwent orthopedic surgeries. Postoperative delirium occurred in 24%. High YKL-40 PREOP and IL-6 at POD2 (Q4 vs. Q1) were significantly associated with an increased risk of delirium: relative risk (RR) [95% confidence interval (CI)] 2.2[1.1-4.4] and 2.7[1.3-5.7], respectively. CRP (PREOP and POD2) was not significantly associated with delirium (p=0.37 and p=0.73, respectively). This work underscores the importance of inflammation (YKL-40 and IL-6) in the pathophysiology of postoperative delirium.

